# Transparenz in der klinischen Forschung: Welchen Beitrag leistet die neue EU-Verordnung 536/2014?

**DOI:** 10.1007/s00103-022-03631-x

**Published:** 2022-12-13

**Authors:** Daniel Strech

**Affiliations:** QUEST Center for Responsible Research, Berlin Institute of Health at Charité – Universitätsmedizin Berlin, Anna-Louisa-Karsch-Str. 2, 10178 Berlin, Deutschland

**Keywords:** Transparenz, EU-Verordnung 536/2014, Klinische Studien, Registrierung, Publikation, Transparency, EU Regulation 536/2014, Clinical trials, Registration, Reporting

## Abstract

Klinische Studien können in 4 Bereichen mehr oder weniger transparent sein: A) Studienregistrierung, B) Ergebnisveröffentlichung, C) Teilen von Daten und Codes, D) studienbezogene Dokumente. Dieser Diskussionsbeitrag erläutert, in welcher Ausprägung die EU-Verordnung 536/2014 (Clinical Trials Regulation – CTR) den Bereich Ergebnisveröffentlichung bei interventionellen Arzneimittelstudien nachweisbar positiv beeinflusst hat und wie sie die Verfügbarkeit studienbezogener Dokumente für unabhängige Forschung in Zukunft verbessern kann.

Da der positive Trend aktuell nur im Bereich Ergebnisveröffentlichung auszumachen ist und zudem nur bei der Subgruppe interventioneller Arzneimittelstudien, scheint sich eine „2-Klassen-Transparenz“ zu entwickeln, die zwischen Studien gemäß CTR und den übrigen Studien unterscheidet. Unabhängig von der CTR sollten sich deshalb akademische Einrichtungen, Förderer und Ethikkommissionen mit der besseren Umsetzung aller 4 Transparenzbereiche beschäftigen und dies bei *allen* klinischen Studien. Ein Monitoring zur Umsetzung der Transparenz bei klinischen Studien wäre ein wichtiger erster Schritt, um den Handlungsbedarf zu konkretisieren. Eine Neuerung im Kontext Transparenz klinischer Studien könnte sich zudem dadurch ergeben, dass das neue EU-Portal „Clinical Trials Information System“ (CTIS) gemäß CTR studienbezogene Dokumente zur informierten Einwilligung (Informed Consent), Studienprotokolle und Prüferinformationen (Investigator Brochures) transparenter machen soll. Damit ergäbe sich erstmals die Möglichkeit einer unabhängigen Forschung und Qualitätssicherung zu Fragen der informierten Einwilligung und der Nutzen-Schaden-Abwägung bei klinischen Studien.

## Abstract (en)

Clinical studies can be more or less transparent in four areas: (a) study registration, (b) results reporting, (c) data/code sharing, and (d) study-related documents. This discussion paper explains the extent to which the EU Regulation 536/2014 (Clinical Trials Regulation – CTR) has already positively impacted the area of results reporting in interventional drug trials and how it can improve the availability of study-related documents for independent research in the future.

As this positive trend exists only for the area of results reporting and for the subset of interventional drug trials addressed by the CTR so far, a problematic two-class transparency seems to be developing that distinguishes between clinical studies addressed by the CTR and the other clinical studies. Independently of the CTR, academic institutions, funders, and ethics committees should therefore address all four abovementioned areas of transparency in *all* clinical studies. Monitoring the implementation of transparency in clinical studies would be an important first step in order to specify the need for action. An innovation in the context of transparency of clinical trials could also arise from the fact that the new EU Portal Clinical Trials Information System (CTIS) according to the CTR makes study-related informed consent documents, study protocols, and the investigator’s brochures more transparent. This would for the first time open up the opportunity of independent research and quality assurance on issues of informed consent and harm–benefit assessment in clinical research.

## Einleitung

Die Transparenz von klinischen Studien, deren Design und deren Ergebnissen wird von allen AkteurInnen der klinischen Forschung als entscheidend angesehen, um den Wert dieser Forschung für die Gesellschaft sicherzustellen und kontinuierlich zu verbessern. Transparenz reduziert Ineffizienzen z. B. durch die Vermeidung von Forschung, die nicht oder anders durchgeführt worden wäre, hätte man zeitnah Informationen über die Existenz von ähnlichen Studien gehabt. Transparenz ist zudem von zentraler Relevanz, um vollständige und damit unverzerrte Informationen über die erwünschten und unerwünschten Effekte medizinsicher Maßnahmen zu erhalten. Unzureichende Transparenz der Ergebnisse klinischer Forschung kann bei Behandelnden und ihren PatientInnen zu Fehlentscheidungen im Zusammenhang mit der Anwendung medizinischer Maßnahmen führen, wodurch wiederum der PatientInnenschutz gefährdet wird. Für weitere Ziele und Mechanismen von Transparenz zu klinischen Studien siehe Tab. 1.

**Table 1:** 

Ziele von Transparenz	Mechanismen zur Zielerreichung
Ermöglichung effizienter Forschung	Wissen über laufende und abgeschlossene Studien und deren Ergebnisse ermöglicht gut informierte Entscheidungen zur Auswahl, zum Design und zur Förderung neuer Studien mit ausreichend hohem Potenzial für einen echten Erkenntnisgewinn
Ermöglichung effektiver Begutachtung klinischer Studien	Ethikkommissionen und Bundesoberbehörden können die Angemessenheit neuer klinischer Studien nur dann kompetent bewerten, wenn sie unverzerrt über die bisherige Studienlage informiert werden
Ermöglichung evidenzbasierter medizinischer Versorgung	Wenn Informationen über Studien, Studiendesigns und deren Ergebnisse nicht vollständig sind, wird damit die Informationsgrundlage (Evidenz) für Entscheidungen in der Leitlinienentwicklung oder direkt in der individuellen Patientenversorgung verzerrt
Ermöglichung der Studiensuche für PatientInnen und ÄrztInnen	Der öffentliche Zugang zu einer vollständigen und aktualisierten Listung laufender und abgeschlossener Studien ermöglicht es PatientInnen, ihren Angehörigen oder ÄrztInnen möglicherweise passende Studien für ihre Erkrankung zu finden
Gesellschaftliches Vertrauen	Vollständige Transparenz zu klinischen Studien und deren Ergebnissen ist eine wichtige Maßnahme, um gesellschaftliches Vertrauen in diesen Forschungsbereich zu generieren und aufrechtzuerhalten

Trotz der Einigkeit über den Bedarf an Transparenz in der klinischen Forschung gibt es anhaltende Kontroversen über deren angemessene Spezifizierung. Wie zeitnah, wie zugänglich (Open Access), in welchem Format (Fachartikel, Summary Results) und in welcher Verbindung zur vorherigen Registrierung der Studie sollten die Ergebnisse veröffentlicht werden, damit sie nicht nur für regulatorische Prozesse, sondern insbesondere auch für die Wissenschaft und die Gesellschaft nützlich sind? Empirische Evaluationen zur Umsetzung „nützlicher“ Transparenz zeigen verschiedene Optimierungspotenziale auf. Das Ziel dieses Beitrages ist es, diese Kontroversen und Umsetzungsfragen für klinische Studien darzustellen, unabhängig davon, ob sie durch die EU-Verordnung 536/2014 (Clinical Trials Regulation – CTR) adressiert werden oder nicht. Zugleich wird die besondere Bedeutung der CTR herausgearbeitet, da sie als gesetzliche Vorgabe zur Transparenz von interventionellen Arzneimittelstudien direkt und indirekt Auswirkungen auf das Verständnis und die Umsetzung von Transparenz bei allen klinischen Studien hat.

Der Fokus dieses Beitrages liegt auf interventionellen klinischen Studien, welche grundsätzlich zur Evaluation aller medizinischen Maßnahmen verwendet werden können. Dazu gehören neben Arzneimitteln auch Medizinprodukte, psychotherapeutische oder chirurgische Verfahren.^1^ Die Transparenz nichtinterventioneller Studien ist aus den oben genannten Gründen genauso wichtig wie die Transparenz zu interventionellen Studien. Es würde allerdings einen eigenen Beitrag benötigen, um die zum Teil sehr grundlegenden Kontroversen zur Binnendifferenzierung von nichtinterventionellen Studien darzustellen (Stichwort: Anwendungsbeobachtungen [1]). Des Weiteren existieren bislang nur sehr wenige Studien, die die Umsetzung von Transparenz bei nichtinterventionellen Studien systematisch untersucht haben [2].

Nach einer Einführung in 4 komplementäre Transparenzbereiche bei klinischen Studien, werden einige empirische Untersuchungen zu deren konkreter Umsetzung präsentiert. Vor diesem Hintergrund wird in 2 folgenden Kapiteln dargestellt, wie sich über die letzten Jahre eine „2-Klassen-Transparenz“ zu entwickeln scheint. Diese ist dadurch gekennzeichnet, dass nur solche Studien transparenter werden, die von der CTR adressiert werden, und dies auch nur in bestimmten Transparenzbereichen, die in der CTR festgelegt werden. Abschließend wird das Potenzial vorgestellt, welches in der Transparenz von studienbezogenen Dokumenten liegt, und es werden Einschränkungen von Transparenz aufgezeigt, die durch unabhängige Forschergruppen evaluiert werden sollten.

## Wie genau wird klinische Forschung transparent?

Grob lassen sich 4 Bereiche unterscheiden, in denen klinische Studien mehr oder weniger transparent sein können:

A. *Studienregistrierung*: Transparenz des Studienvorhabens, wie z. B. geplanter Einschlusskriterien, Endpunkte, Laufzeiten, Statistik [3–7];
B. *Ergebnisveröffentlichung*: Transparenz der de facto untersuchten Population der Studienteilnehmenden und zu den Studienergebnissen [3, 5–8];
C. *Teilen von Daten und Codes (Data‑/Code Sharing)*: Transparenz der nicht aggregierten Daten und zu den verwendeten Algorithmen/Codes zur Auswertung der Daten [6, 9, 10];
D. *studienbezogene Dokumente*: Transparenz zu z. B. Studienprotokollen, Prüferinformationen (Investigator Brochures – IB), Ethikvoten oder Texten zur Aufklärung und Einwilligung [7, 11].

Für alle 4 Transparenzbereiche gibt es spezifische Anforderungen:

A. Die Registrierung von Studien sollte prospektiv, also vor dem Studienstart, stattfinden und im Studienverlauf aktualisiert werden (wenn z. B. eine Studie länger oder kürzer läuft, Endpunkte geändert werden etc.; [12]). Bei einer Registrierung in verschiedenen Registern sollten die Informationen konsistent sein und es sollte auf die jeweils andere Registrierung verwiesen werden [13].
B. Die Ergebnisveröffentlichungen als Fachartikel sollten zeitnah erfolgen (innerhalb von 2 Jahren nach Studienende; [8]), die Registrierungsnummer angeben [14] und nicht nur hinter einer Bezahlschranke erreichbar sein (Open Access). Unabhängig von Fachartikeln sollten Zusammenfassungen der Ergebnisse (Summary Results) auf der jeweiligen Registerseite veröffentlicht werden.
C. Das Teilen von Daten und Codes sollte in einem Data-Sharing-Plan im Registereintrag der Studie beschrieben und in einem Data-Sharing-Statement in der Publikation genannt werden [15]. Das Teilen von Daten und Codes sollte den FAIR-Prinzipien entsprechen (Findability, Accessibility, Interoperability, Reusability; [16]).
D. Für studienbezogene Dokumente, wie die oben genannten, bestehen in der Regel Vorgaben und national/international etablierte Empfehlungen zur Sicherstellung einer ausreichenden Informationsqualität, z. B. bei Investigator Brochures [17] oder Dokumenten zur informierten Einwilligung (Informed Consent – IC; [18]).

Wie durch die Referenzen im obigen Text aufgezeigt, liegen zu diesen 4 Transparenzbereichen national und international renommierte (ethische) Leitlinien und Empfehlungen vor. Im Folgenden werden Herausforderungen und damit verbundene Bedarfe rund um das Thema „mehr Transparenz“ vorgestellt.

## Trends in der Umsetzung von Transparenzvorgaben

Bestimmte Transparenzvorgaben wurden über die vergangenen Jahre zunehmend umgesetzt. Die Entwicklungen über die letzten 2–3 Jahre zeigen z. B. einen starken Anstieg der Ergebnisveröffentlichung der von deutschen medizinischen Fakultäten verantworteten klinischen Studien, die im EU Clinical Trials Register (EUCTR) registriert und damit von der CTR adressiert sind.^2^ Im Jahr 2019 wurden laut einem Bericht der Organisation „TranspariMED“ auf der Basis von Daten des EU Trials Tracker^3^ der Universität Oxford, Vereinigtes Königreich, durchschnittlich für weniger als 5 % der Studien medizinischer Fakultäten die Ergebnisse im EUCTR (als Summary Results) veröffentlicht [19]. Die Charité – Universitätsmedizin Berlin, die die größte Anzahl an klinischen Studien in Deutschland zu verantworten hat, hatte zu diesem Zeitpunkt bei nur 2 % ihrer im EUCTR registrierten Studien die Vorgaben zur Ergebnisveröffentlichung gemäß CTR erfüllt.

Infolge des u. a. durch diese Evaluationen gestiegenen öffentlichen und politisch-behördlichen Drucks über die letzten 2–3 Jahre nahm die Ergebnisveröffentlichung für diese unter die EU-Gesetzgebung fallenden Studien stark zu. Laut EU Trials Tracker (Stand 26.08.2022) liegt die Ergebnisveröffentlichung (als Summary Results im EUCTR) der Charité – Universitätsmedizin Berlin nun bei 95 %. Auch wenn aktuell noch nicht alle deutschen Universitäten diese hohe Umsetzungsrate erreicht haben, ist der positive Trend über alle deutschen Universitäten hinweg sehr stark. Klinische Studien von kommerziellen Sponsoren hatten übrigens i. d. R. bereits im Jahr 2019 hohe Raten an Summary Results Reporting [20].

Anders als bei den im EUCTR registrierten Studien deutscher Universitäten hat sich die Veröffentlichung von Summary Results für klinische Studien, die in den Registern „clinicaltrials.gov“ oder DRKS (Deutsches Register Klinischer Studien) registriert sind, nicht verbessert. Weiterhin werden in deutlich weniger als 10 % der Fälle Studienergebnisse als Summary Results in clinicaltrials.gov oder DRKS berichtet [21]. Das könnte auf den ersten Blick daran liegen, dass das Thema Summary Results bzw. die Ergebnisveröffentlichung in Registern für viele Studienverantwortliche noch relativ neu ist. Die Publikation eines Fachartikels mag für die meisten Studienverantwortlichen weiterhin der primäre Weg für Ergebnisveröffentlichungen sein. Allerdings konnte eine Nachverfolgung aller ca. 1600 klinischen Studien deutscher Universitäten, die in clinicaltrials.gov oder DRKS registriert sind und zwischen 2014 und 2017 abgeschlossen wurden, nur bei 43 % eine zeitnahe Ergebnisveröffentlichung (2 Jahre nach Studienende) als Fachartikel finden [21]. Auch 5 Jahre nach Studienende hatten 30 % aller Studien noch keine Ergebnisse veröffentlicht (weder als Fachartikel noch als Summary Results). Die CTR hilft an dieser Stelle nicht weiter, weil sie für die in anderen Registern registrierten Studien nicht bindend ist.

Neben der aus medizinischer und ethischer Perspektive zentralen Frage, ob die Studienergebnisse in irgendeiner nützlichen Form veröffentlicht werden, ist es zur Förderung des Wertes und der Effizienz klinischer Studien auch wichtig, dass die oben genannten spezifischen Transparenzanforderungen zur Registrierung und Ergebnisveröffentlichung umgesetzt werden, wie z. B. die Verlinkung zwischen Registrierung und korrespondierenden Fachartikeln. Gegenwärtig nennen nur ca. 40 % der Fachartikel von in clinicaltrials.gov oder DRKS registrierten Studien deutscher Universitäten ihre Registernummer [22]. Auch wird ein vorhandener Fachartikel in nur ca. 45 % der Fälle im Register verlinkt [22]. Dabei würde die Umsetzung dieser Transparenzempfehlungen kaum Zeit kosten.

Nicht zuletzt sind auch die Open-Access-Raten bei Fachartikeln zu klinischen Studien ein wichtiger Transparenzaspekt. Open-Access-Gebühren von oft 2000–4000 € sind eine Herausforderung für viele biomedizinische Studien. Angesichts der i. d. R. viele 100.000 € teuren klinischen Studien sollten die Gebühren aber eigentlich kein Grund dafür sein, nicht Open Access zu publizieren und damit für PatientInnen oder ÄrztInnen, die regulär keinen Zugang zu Universitätsbibliotheken haben, nicht bzw. nur hinter einer Bezahlschranke (Paywall) zur Verfügung zu stehen. Während die Open-Access-Rate von Publikationen zu klinischen Studien bei Artikeln, die im Jahr 2010 publiziert wurden, ca. 25 % betrug, sind von den im Jahr 2020 veröffentlichten Artikeln bereits ca. 65 % Open Access verfügbar (unveröffentlichte Daten, in Begutachtung). Allerdings könnte die große Mehrzahl der älteren und neuen veröffentlichten Artikel ohne Open Access relativ einfach über Repositorien wie „Zenodo“^4^ oder universitätseigene Repositorien öffentlich verfügbar gemacht werden (sogenanntes Green Open Access). Hierfür werden die Versionen der Fachartikel verwendet, die zur Publikation angenommen wurden, die aber noch nicht in das Layout der Zeitschrift überführt wurden. Ähnlich wie bei dem Beispiel für Summary Results könnten auch bei Open Access auf diesem Wege schnell Open-Access-Raten von über 95 % erreicht werden. Leider setzt nur ein sehr geringer Anteil der AutorInnen von Fachartikeln zu klinischen Studien diese einfache und kostenlose Option für ein Open Access ihrer Beiträge um.

## Die EU-Gesetzgebung adressiert nur eine Subgruppe aller in Deutschland durchgeführten klinischen Studien

So positiv die jüngeren Entwicklungen zur hohen Rate an Veröffentlichungen von Summary Results im EUCTR sind, so deutlich wird auch, dass durch die Vorgaben für Arzneimittelstudien in der CTR (ungewollt) eine Art von „2-Klassen-Transparenz“ generiert wird. Fällt eine interventionelle Studie nicht unter die EU-Gesetzgebung für Arzneimittel, gibt es keine gesetzliche Pflicht, diese Studie im EU-Register zu registrieren und deren Ergebnisse als Summary Results im Register zu berichten (siehe oben). Zwar sieht die EU-Gesetzgebung zu Medizinprodukten (EU-Verordnung 2017/745) ebenfalls eine Registrierung und Ergebnisveröffentlichung für interventionelle Studien zu Medizinprodukten vor, die in einer überarbeiteten eigenen Datenbank (Europäische Datenbank für Medizinprodukte – Eudamed) stattfinden soll; allerdings soll diese initial für 2020 geplante Datenbank neuesten Angaben zufolge erst im Jahr 2024 starten [23]. Für interventionelle Studien zu anderen medizinischen Maßnahmen wie Psychotherapie oder Chirurgie gibt es keine entsprechenden gesetzlichen Regelungen.

Die Logik hinter transparenter Forschung zum Nutzen der Gesellschaft, zur Vermeidung von Ineffizienzen und zum PatientInnenschutz trifft aber auf alle klinischen Studien gleichermaßen zu. Deshalb richten sich auch international etablierte Transparenzempfehlungen an alle klinischen Studien, unabhängig davon, ob sie Arzneimittel oder andere medizinische Interventionen untersuchen. Zentral sind dabei z. B. die Prinzipien 35 und 36 der Deklaration von Helsinki, welche die Registrierung und Ergebnisveröffentlichung einfordern und zwar für alle klinischen Studien oder gemäß der Deklaration von Helsinki noch allgemeiner für jede „Forschung am Menschen“ [3]. Auch andere international etablierte Transparenzleitlinien wie die Empfehlung des International Committee of Medical Journal Editors (ICMJE) zur prospektiven Registrierung [4] oder die Empfehlungen der Weltgesundheitsorganisation (WHO) zur zeitnahen Ergebnisveröffentlichung innerhalb von 2 Jahren [8] adressieren explizit alle klinischen Studien. Auch die Senatskommission für Grundsatzfragen in der Klinischen Forschung (SGKF) der Deutschen Forschungsgemeinschaft (DFG) sprach sich in einer Stellungnahme im Jahr 2018 für eine prospektive Registrierung und eine zeitnahe Ergebnisveröffentlichung aller „klinischen Studien“ aus [5].

## Wie viele der in Deutschland durchgeführten interventionellen Studien sind nicht von der EU-Verordnung 536/2014 (CTR) adressiert?

Es ist mit öffentlich verfügbaren Quellen nicht einfach zu beziffern, wie groß die Gruppe der in Deutschland durchgeführten interventionellen Studien ist, die nicht von der EU-Gesetzgebung adressiert ist. Die am ehesten hilfreichen, aber trotzdem für diese Frage stark eingeschränkten Quellen sind die nationalen und internationalen Studienregister. Das EUCTR gibt an, dass in den Jahren 2016 bis 2020 in Deutschland pro Jahr durchschnittlich etwa 1000 neue interventionelle Arzneimittelstudien gestartet sind. Diese Zahl deckt sich erwartungsgemäß mit den Angaben des Bundesinstituts für Arzneimittel und Medizinprodukte (BfArM) und des Paul-Ehrlich-Instituts (PEI), die für diesen Zeitraum auf ihren Webseiten zusammengenommen ebenfalls jährlich ca. 1000 Genehmigungen zu interventionellen Arzneimittelstudien („klinische Prüfungen“) angeben. Das Register clinicaltrials.gov gibt für denselben Zeitraum für Deutschland auch etwa 1000 interventionelle Studien pro Jahr an, das DRKS etwa 700 (übergreifend für Arzneimittel, Medizinprodukte und andere medizinische Maßnahmen). Einige dieser in clinicaltrials.gov und DRKS registrierten Studien könnten Zweitregistrierungen von bereits im EUCTR registrierten Studien sein. Da aber viele der in clinicaltrials.gov oder im DRKS registrierten Studien keine Arzneimittelstudien sind, dürfte davon auszugehen sein, dass mehrere Hundert interventionelle Studien jährlich in Deutschland stattfinden, die nicht im EUCTR registriert sind.

Da alle klinischen Studien von einer Ethikkommission begutachtet werden müssen, könnte eine zentrale Sammelstelle für Entscheidungen der Ethikkommissionen prinzipiell angeben, wie viele und welche klinischen Studien in Deutschland genehmigt wurden. Nach Anfrage beim Arbeitskreis Medizinischer Ethik-Kommissionen (AKEK) wurde bestätigt, dass die Anzahl begutachteter Anträge auf freiwilliger Basis von den einzelnen Ethikkommissionen abgefragt wird. Um die Gesamtzahl aller begutachteten klinischen Studien zu erfassen, inklusive der nicht nach Vorgaben des Arzneimittelgesetzes (AMG) und Medizinproduktegesetzes (MPG), sondern nach Berufsrecht begutachteten Studien, müssten aber noch weitere Klärungen erfolgen. Die Anzahl der nach Berufsrecht begutachteten Forschungsprojekte, die neben interventionellen Studien zu Themen außerhalb des AMG oder MPG allerdings auch Umfragen und andere nichtklinische Studien umfassen können, ist nach Auskunft vom AKEK deutlich höher als die Anzahl der nach AMG/MPG begutachteten klinischen Studien.

## Die EU-Gesetzgebung adressiert nur einen Teil der international etablierten Transparenzempfehlungen

Die „2-Klassen-Transparenz“, die im vorausgegangenen Kapitel beschrieben wurde, bezog sich auf bestimmte Gruppen interventioneller Studien: Die (Arzneimittel‑)Studien, die von der CTR adressiert werden, konnten einen positiven Trend bei der Ergebnisveröffentlichung verbuchen, aber andere interventionelle Studien jedoch nicht. Eine weitere Form von „2-Klassen-Transparenz“ kann für die 4 oben beschriebenen Transparenzbereiche und deren spezielle Anforderungen beobachtet werden. Nur in den Transparenzbereichen, die von der CTR adressiert werden (hier speziell die Veröffentlichung von Summary Results), lassen sich klare Verbesserungen ausmachen. Andere Bereiche bzw. deren spezielle Anforderungen, wie z. B. die Publikation von Fachartikeln, die Nennung der Registernummer im Fachartikel oder Open Access, die alle nicht Teil der Verordnung sind, haben sich über die letzten Jahre deutlich weniger bis kaum verbessert (siehe oben).

Auch der dritte Transparenzbereich, das Data‑/Codes Sharing, ist nicht explizit Teil der EU-Gesetzgebung. Die ICMJE-Vorgaben zu Data Sharing fordern für alle klinischen Studien mit Studienstart ab 2019, dass ein Data-Sharing-Plan mit dem Registereintrag verlinkt wird. In clinicaltrials.gov ist dies möglich, im EUCTR bislang nicht. Prinzipiell wäre es sinnvoll, wenn es zukünftig im CTIS die Möglichkeit geben würde, unter der Rubrik „Documents“ ein zusätzliches Dokument wie den Data-Sharing-Plan hochzuladen. Ein bestimmtes Feld für einen Data-Sharing-Plan ist aber nach Wissen des Autors im CTIS aktuell nicht vorgesehen.

All die hier genannten Aspekte von Transparenz wie Ergebnisveröffentlichungen als Fachartikel, die Verlinkung zwischen Registrierung und Fachartikel, Open Access oder Data Sharing müssen natürlich nicht notwendigerweise in einem Gesetz gefordert werden. Es wäre zu begrüßen, wenn sogenannte Soft Laws (nicht verbindliche Übereinkünfte, Absichtserklärungen oder Leitlinien) zum Thema Transparenz, wie die Deklaration von Helsinki oder Empfehlungen der WHO, des ICMJE oder der DFG, ausreichend berücksichtigt würden.

## Transparenz zu studienspezifischen Dokumenten

Die CTR benennt als eines ihrer Hauptziele die Harmonisierung und Stärkung von Transparenz [24]. Wie oben beschrieben, ist mit der Stärkung jedoch keine inhaltliche Ausweitung der ersten 3 Transparenzbereiche gemeint. Der vierte Transparenzbereich (studienspezifische Dokumente) wird jedoch auch inhaltlich gestärkt (zumindest theoretisch). So sollen in Zukunft nicht nur die studienspezifischen Daten, sondern auch relevante Dokumente mit dem Registereintrag verlinkt werden. In der Interpretation verschiedener KommentatorInnen fallen unter diese Dokumente neben dem Studienprotokoll und dem Clinical Study Report (CSR) auch Aufklärungsmaterialien (IC-Dokumente) und die Investigator Brochures [25]. Je nach Umsetzung dieser Vorgaben könnte ein sehr hohes Nutzenpotenzial für unabhängige Forschungsgruppen entstehen. Dies soll an 2 Beispielen skizziert werden.

Die Qualität der Inhalte und die Verständlichkeit von Aufklärungsmaterialien zu klinischen Studien sind schwer zu beforschen, da diese häufig nicht öffentlich und nicht in enger Verknüpfung mit dem jeweiligen Studienprotokoll oder der Investigator Brochure verfügbar sind. Die bisherige Forschung zu IC-Dokumenten ist deshalb nur möglich, wenn SponsorInnen oder StudienärztInnen direkt angeschrieben und um Zusendung von IC-Dokumenten gebeten werden [26, 27]. Die Responserate ist dabei niedrig (15–30 %). Wenn IC-Dokumente durch die CTR regelmäßiger im Register verfügbar werden, kann die Qualitätssicherung durch unabhängige Forschungsgruppen profitieren. Von Informationen über den Status quo zur Qualität von IC-Dokumenten und zu Optimierungsmöglichkeiten kann die klinische Forschung selber und können konsekutiv auch die Studienteilnehmenden profitieren.

Gegenwärtig ebenfalls nur sehr schwer unabhängig zu beforschen sind die Investigator Brochures, die als Informationsgrundlage für Abwägungen von Nutzen- und Schadenspotenzialen im Begutachtungsverfahren klinischer Studien dienen. Solche Forschung kann aktuell nur in sehr limitierter Form z. B. in Kooperation mit wenigen Ethikkommissionen durchgeführt werden, die den Zugang zu den Investigator Brochures unter strengen Vertraulichkeitsvorgaben ermöglichen können. Eine in Deutschland durchgeführte Studie anhand von 100 Investigator Brochures für Phase-I/II-Studien lieferte jedoch wichtige Erkenntnisse zu substantiellen Limitationen der Abwägungsprozesse im Begutachtungsverfahren früher klinischer Forschung [28, 29]. Verschiedene Fragen zur Optimierung der Informationsqualität entsprechender Dokumente wären erst möglich, wenn deren Transparenz verbessert würde [30].

## Möglichkeiten eingeschränkter oder verzögerter Transparenz durch die EU-Gesetzgebung

Bei den oben genannten Transparenzvorgaben bzgl. Ergebnisveröffentlichung wie auch bei den relevanten Dokumenten und Daten ermöglicht die CTR in Artikel 81 Ausnahmen für einzelne Studien, die genehmigt werden können, wenn sie einen oder mehrere der folgenden Punkte ausreichend adressieren:

- Schutz personenbezogener Daten gemäß der Verordnung (EG) Nr. 45/2001,
- Schutz von Betriebs- oder Geschäftsgeheimnissen, insbesondere durch Berücksichtigung des Status der Zulassung des Arzneimittels, sofern kein übergeordnetes öffentliches Interesse an der Offenlegung besteht,
- Schutz vertraulicher Mitteilungen zwischen Mitgliedstaaten bezüglich der Ausarbeitung des Bewertungsberichts,
- Gewährleistung einer wirksamen Überwachung der Durchführung der klinischen Prüfung durch die Mitgliedstaaten.

Weiterhin erlaubt die CTR bei Studien mit Erwachsenen auch eine Verzögerung (*Deferral*) der Veröffentlichung von Ergebnissen und Dokumenten um bis zu 7 Jahre, wenn diese mit Bezug auf konfligierende kommerzielle Aspekte ausreichend begründet werden [31]. Diese Gründe müssen ebenfalls transparent gemacht werden. Eine Listung von Gründen, die als ausreichend gelten und das öffentliche Interesse an Transparenz überwiegen, findet sich in den dem Autor bekannten Dokumenten der EU bzw. der Europäischen Arzneimittel-Agentur (EMA) nicht.

Wie umfassend von der Ausnahmeregelung und der zeitlichen Verzögerung bei den Transparenzvorgaben Gebrauch gemacht wird, lässt sich wenige Monate nach Start des CTIS mit den i. d. R. gerade erst begonnenen Studien noch nicht bewerten. Mit dieser Frage sollten sich unabhängige Forschungsgruppen in den nächsten Jahren intensiver beschäftigen.

## Was würde die (unabhängige) Forschung über klinische Forschung stärken?

Die in diesem Beitrag dargestellten Herausforderungen und Verbesserungsmöglichkeiten rund um das Thema Transparenz bei klinischen Studien lassen folgende Schlussfolgerungen zu.

Die Akteure der klinischen Forschung (Forschende, akademische Einrichtungen, Förderer, Behörden, Ethikkommissionen) sollten sich noch stärker bewusstwerden, dass die gesetzlichen Vorgaben der CTR notwendig, aber nicht hinreichend für einen verantwortungsvollen Umgang mit Transparenz sind. Zum einen werden viele interventionelle klinische Studien in Deutschland von dieser Verordnung nicht adressiert. Zum anderen adressiert die Verordnung nur einen kleinen Teil der international etablierten Anforderungen an die Transparenz klinischer Studien.

Die zunehmende Veröffentlichung von Studienergebnissen als Summary Results im EUCTR (bald im CTIS) wurde als eine positive Entwicklung herausgearbeitet. Solange aber Fachartikel weiterhin als ergänzende oder bei Studien außerhalb der CTR auch als alleinige Form der Ergebnisveröffentlichung verwendet werden, sollten die damit einhergehenden Transparenzgebote stärker beachtet werden. Dazu gehören die zeitnahe Veröffentlichung [8], die Nennung der Registernummer [14], die Verlinkung mit dem Registereintrag und Open Access.

Transparenzvorgaben, ob von der CTR, der Deklaration von Helsinki, des ICMJE oder weitere, sind nur der erste Schritt. Die Akteure der klinischen Forschung sollten die Umsetzung der Transparenzvorgaben monitoren und bei unzureichender Umsetzung, die Praxis mit Information, Unterstützung und Anreizen verbessern. Während die Behörden aufgrund ihres Aufgabenbereichs nur die Umsetzung von Transparenz bei solchen Studien beeinflussen können, die durch die CTR oder AMG/MPG adressiert werden, sind die weiteren Akteure wie akademische Einrichtungen, Förderer und Ethikkommissionen gefragt, die Transparenzumsetzung auch bei allen anderen klinischen Studien zu fördern. Ein unabhängiges Tool zum Monitoring des Summary Results Reporting der von der CTR adressierten Studien bietet der bereits erwähnte EU Trials Tracker. Wie oben beschrieben, ist damit aber nur eine Subgruppe aller klinischen Studien abgebildet und es wird nur eine spezielle Transparenzvorgabe evaluiert (das Berichten von Summary Results). An weiteren Tools, die zum einen alle (interventionellen) klinischen Studien berücksichtigen und zum anderen weitere Transparenzbereiche adressieren, wird gegenwärtig gearbeitet. Es werden Datensätze zur Transparenz der in Deutschland durchgeführten klinischen Studien erstellt [21, 32]. Diese Datensätze können in nächsten Schritten u. a. dazu verwendet werden, Dashboards zu erstellen, welche die Transparenz bei klinischen Studien für einzelne medizinische Fakultäten darstellen [33].^5^ In einem weiteren Ansatz soll über sogenannte Report Cards die Transparenzperformance für einzelne Studien an die Studienverantwortlichen zurückgemeldet werden, zusammen mit konkreten Hinweisen, wie die einzelnen Transparenzbereiche verbessert werden können [34].

Die Forderung der CTR, auch studienspezifische Dokumente wie IC-Dokumente oder die Investigator Brochures transparent zu machen, würde die unabhängige Forschung zu weiteren Aspekten verantwortungsvoller klinischer Forschung ermöglichen. Es wäre dabei wichtig, dass nicht zu umfangreich von den Ausnahmeregelungen (Art. 81) und Verzögerungen (Deferrals) Gebrauch gemacht wird, die ebenfalls durch die CTR ermöglicht werden.

## Footnotes

1 Unter dem Überbegriff „klinische Studien“ (englisch: „clinical studies“) werden international interventionelle und nichtinterventionelle Studien verstanden. Siehe z. B. das Glossar des international etablierten Registers clinicaltrials.gov (https://clinicaltrials.gov/ct2/about-studies/glossary). Interventionelle Studien werden im Englischen synonym als „clinical trials“ und nichtinterventionelle Studien als „observational studies“ bezeichnet. Im Deutschen wird für interventionelle Studien gemäß Arzneimittelgesetz (AMG) und Medizinproduktegesetz (MPG) der Begriff der „klinischen Prüfung“ verwendet. Bei interventionellen Studien zu anderen medizinischen Interventionen, wie z. B. Psychotherapie oder Chirurgie, ist der Begriff der klinischen Prüfung hingegen nicht etabliert. Um Missverständnisse zu vermeiden, verwendet dieser Beitrag deshalb primär den Begriff interventionelle Studie (und nicht klinische Prüfung), um damit alle medizinischen Interventionen zu adressieren.

2 Das bisherige EUCTR wird über die kommenden 3 Jahre schrittweise von dem neuen CTIS (Clinical Trials Information System) abgelöst.

3 https://eu.trialstracker.net/.

4 https://zenodo.org/.

5 Siehe als Prototyp z. B. ein Dashboard der Charité – Universitätsmedizin Berlin mit einigen ersten Zahlen zur Transparenz bei klinischen Studien: https://quest-dashboard.charite.de/#tabStart.
